# The Role of Antinuclear Antibody in Predicting the Efficacy and Immune-Related Adverse Events of Immunochemotherapy in Esophageal Squamous Cell Carcinoma: A Real-World Analysis

**DOI:** 10.3390/curroncol33100596

**Published:** 2026-10-01

**Authors:** Ansu Li, Yue Tao, Kongcheng Wang, Haifei Yang, Jiayao Yan, Wu Sun, Wei Ren

**Affiliations:** 1 Department of Clinical Laboratory, Nanjing Drum Tower Hospital, Affiliated Hospital of Medical School, Nanjing University, Nanjing 210008, China; 2 Department of Oncology, Nanjing Drum Tower Hospital, Affiliated Hospital of Medical School, Nanjing University, Nanjing 210008, China

**Keywords:** esophageal squamous cell carcinoma, antinuclear antibody, programmed cell death protein 1, immune-related adverse events, safety, efficacy

## Abstract

**Background:** Programmed cell death protein 1 (PD-1) inhibitor-based immunochemotherapy is a standard treatment for advanced esophageal squamous cell carcinoma (ESCC), but accessible blood-based biomarkers associated with both treatment efficacy and the risk of immune-related adverse events (irAEs) remain limited. The presence of antinuclear antibody (ANA) in the pretreatment period may reflect host immune status; however, its clinical relevance in ESCC and its relationship with programmed death-ligand 1 (PD-L1) expression have not been fully characterized. **Methods:** We retrospectively enrolled 180 patients with ESCC who received PD-1 inhibitor-based immunochemotherapy. Baseline characteristics, tumor responses, survival outcomes, and irAEs were evaluated according to pretreatment ANA status. An additional sensitivity analysis was conducted among 103 patients with available PD-L1 results. Cox regression models with time-varying coefficients were applied when the proportional hazards assumption was not satisfied. **Results:** The ANA-positive rate was 36.67% (66/180). Compared to patients who were ANA-negative, those who were ANA-positive demonstrated superior objective response rates (71.21% vs. 46.49%, *p* = 0.002), longer median progression-free survival (PFS; 11.93 vs. 5.93 months) and longer median overall survival (OS; 25.20 vs. 14.77 months, both *p* < 0.01). In addition, they had a higher rate of all-grade irAEs (60.61% vs. 35.96%, *p* = 0.001), especially thyroid dysfunction (28.79% vs. 8.77%, *p* < 0.001). Multivariable time-varying Cox models linked ANA positivity to favorable PFS (hazard ratio [HR] = 0.14, 95% confidence interval [CI]: 0.04–0.46, *p* = 0.001) and OS (HR = 0.06, 95% CI: 0.01–0.52, *p* = 0.011). In the sensitivity cohort, ANA remained predictive for both survival endpoints, while PD-L1 did not. **Conclusions:** Pretreatment ANA positivity was associated with higher response rates, longer survival, and a higher incidence of irAEs in patients with ESCC receiving PD-1 inhibitor-based immunochemotherapy. These exploratory findings require prospective validation before ANA can be used to guide treatment decisions or assess individual irAE risk.

## Simple Summary

The prevailing treatment protocol for advanced and metastatic esophageal squamous cell carcinoma (ESCC) involves the administration of an anti-PD-1 agent in conjunction with chemotherapy. However, there remains a paucity of biomarkers that reliably predict therapeutic efficacy and the risk of related adverse events. In this study, we identified baseline antinuclear antibodies (ANAs) as a potential circulating biomarker for the concurrent evaluation of therapeutic efficacy and the risk of immune-related adverse events in patients with ESCC undergoing immunochemotherapy. Notably, this prognostic indicator operates independently of PD-L1 status. These findings may enhance the ability to predict the efficacy of immunochemotherapy and facilitate the monitoring of associated toxicities.

## 1. Introduction

Globally, esophageal cancer (EC) ranks eleventh in cancer incidence and seventh in cancer-related mortality worldwide [1]. In China, EC ranks as the seventh most prevalent cancer and the fifth leading cause of cancer-related mortality. Esophageal squamous cell carcinoma (ESCC) represents the predominant histological subtype of EC, accounting for more than 90% of cases [2,3]. ESCC is associated with poor clinical outcome, with 5-year survival rate of ESCC patients only ranging from 20% to 30% across all stages [4]. In recent years, several randomized controlled trials have shown that adding programmed cell death protein 1 (PD-1) inhibitors to chemotherapy can improve clinical outcomes and provide sustained survival benefits in patients with advanced ESCC [5,6,7]. Consequently, PD-1 inhibitor-based immunochemotherapy has become a standard first-line treatment for advanced disease.

Biomarkers like programmed death-ligand 1 (PD-L1) expression, microsatellite instability, and tumor mutational burden have been evaluated to guide cancer immunotherapy. Although these markers may be clinically informative in selected populations, their assessment generally depends on tumor tissue obtained through invasive procedures. Moreover, they do not fully explain the variability in treatment response or reliably identify patients who are likely to develop immune-related adverse events (irAEs). Circulating indicators of host immune status may therefore provide a convenient and minimally invasive alternative [8]. Because the host immune environment can influence the activity of immune checkpoint inhibitors (ICIs), readily measurable serological markers may have substantial value in clinical decision-making [9].

Antinuclear antibody (ANA) is routinely used as a serological marker of autoimmune activity. Although previous studies have examined the associations between ANA status and the efficacy and toxicity of ICI, particularly in non-small-cell lung cancer, the existing evidence remains controversial [10,11]. IrAEs are a prevalent and clinically challenging complication during ICI treatment, which highlights an urgent demand for biomarkers to screen patients at high irAE risk [12,13]. Pretreatment ANA status may reflect the underlying host immune milieu and could therefore be associated with both treatment outcomes and susceptibility to irAEs [14]. Accordingly, this study investigated the associations of pretreatment ANA status with clinical outcomes and irAEs in ESCC patients receiving ICI-based therapy.

## 2. Methods

### 2.1. Study Design and Patients

**Figure 1:**
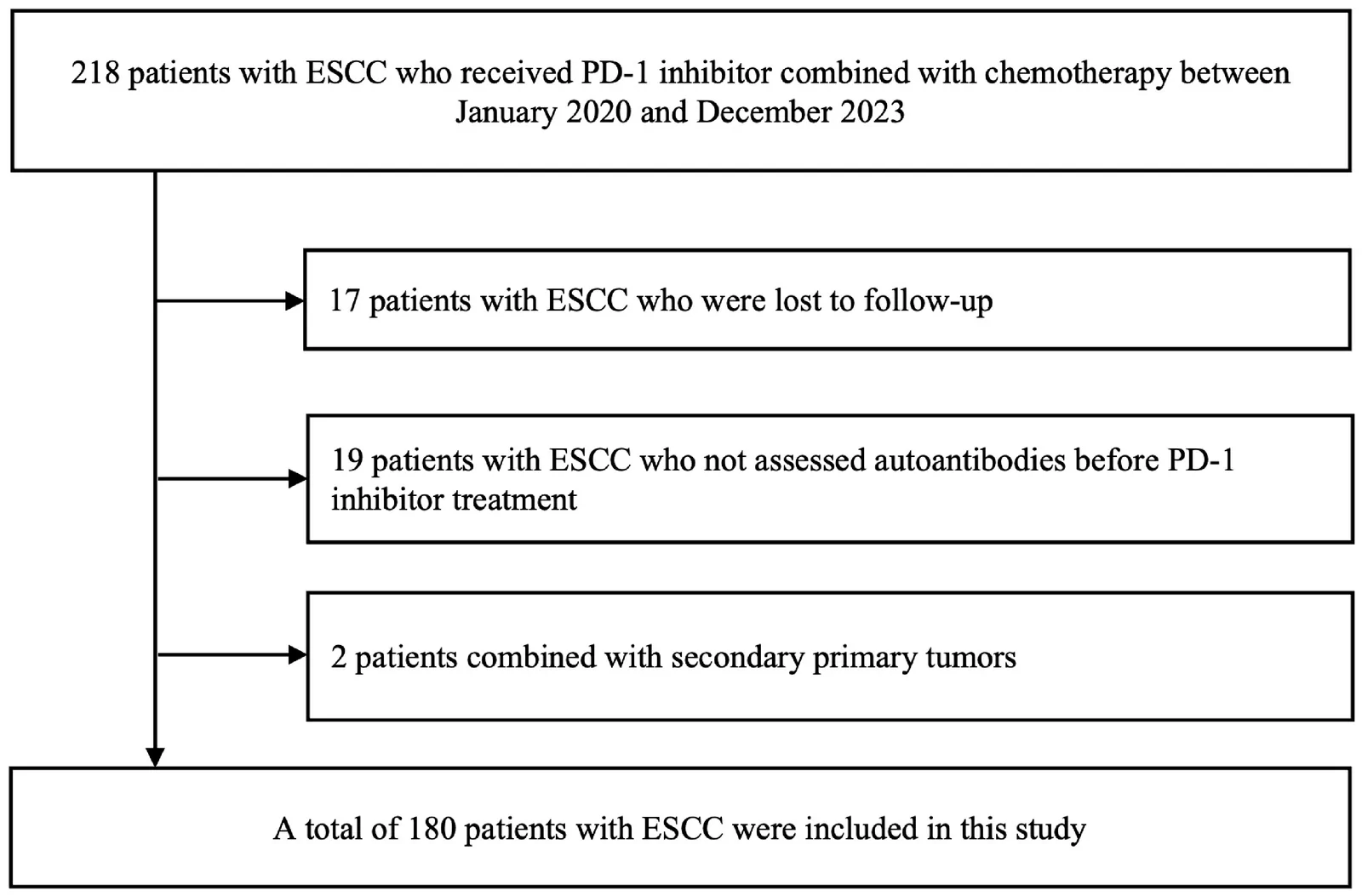
Flowchart of the patient selection process.

This retrospective cohort included patients treated at the Comprehensive Cancer Center of Nanjing Drum Tower Hospital between January 2020 and December 2023. Eligible patients were required to have histopathologically confirmed ESCC, to have received at least one cycle of PD-1 inhibitor-based immunochemotherapy, and to have available pretreatment ANA results. Patients were excluded if they were lost to follow-up, lacked evaluable efficacy or safety data, or had been diagnosed with another malignancy. After eligibility screening, 180 patients were included in the study (Figure 1).

This retrospective cohort study received approval from the Ethics Committee of Nanjing Drum Tower Hospital (No. 2025-0226-01) and was conducted in accordance with the principles outlined in the Declaration of Helsinki.

Demographic and clinical data were retrieved from medical records. Pretreatment variables included age, sex, PD-L1 expression, ANA status, and ECOG performance status. PD-L1 expression was classified by CPS as <10, ≥10, or unknown. We also recorded histological differentiation, disease status, primary tumor location, hepatic metastasis, pulmonary metastasis, the number of metastatic sites, overall autoantibody status and anti-Ro52 antibody status. Additionally, the line of PD-1 inhibitor therapy and the incidence of irAEs were documented.

TNM information was recorded separately for patients without prior surgery and those with postoperative recurrence. For patients without prior surgery, clinical T, N, and M categories and clinical stage groups were recorded before initiation of the index PD-1 inhibitor-based immunochemotherapy regimen. For patients with postoperative recurrence, pathological T and N categories at the original surgery were reported separately. All 62 patients in this group experienced recurrence or metastatic disease after surgery.

The PD-1 inhibitors administered in this cohort were camrelizumab, tislelizumab, toripalimab, and sintilimab. Chemotherapy consisted of nab-paclitaxel combined with carboplatin, lobaplatin, nedaplatin, or cisplatin. Overall treatment distributions are summarized in Table 1, and distributions according to pretreatment ANA status are presented in Supplementary Table S9. The index immunochemotherapy regimens were generally planned in 3-week cycles. Reliable patient-level counts of the PD-1 inhibitor and chemotherapy cycles actually administered could not be determined from the available retrospective data.

### 2.2. Assessments

PD-L1 expression was tested with the DAKO 22C3 pharmDx assay on a Link48 autostainer and scored by combined positive score (CPS). In the present study, a CPS of ≥10 was defined as PD-L1 positivity [15].

Peripheral blood was obtained within 7 days before the first administration of PD-1 inhibitor therapy. Autoantibodies were measured using an indirect immunofluorescence assay (Euroimmun, Lübeck, Germany) according to the manufacturer’s instructions. The testing panel comprised antinuclear antibodies (ANAs) and antibodies targeting Ro52, SSA, SSB, double-stranded DNA (dsDNA), Smith antigen (Sm), ribonucleoprotein (rRNP), U1 ribonucleoprotein (U1RNP), Scl-70, PM-Scl, Jo-1, centromere protein B (CENP-B), proliferating cell nuclear antigen (PCNA), nucleosomes, antimitochondrial antibody M2 (AMA-M2), and histones. ANA positivity was defined as a titer of at least 1:100. ANA analyses were based on pretreatment positivity status; analyses according to individual ANA immunofluorescence patterns were not performed. Baseline autoantibody positivity was defined as the detection of one or more autoantibodies, including ANA, before treatment. As ANA status overlapped substantially with overall autoantibody status, the two variables were entered into separate multivariable models.

The efficacy and safety endpoints were evaluated in the entire cohort of enrolled patients. The efficacy outcomes comprised the objective response rate (ORR), disease control rate (DCR), progression-free survival (PFS), and overall survival (OS). Safety outcomes focused on the occurrence and severity of irAEs. All irAEs were graded according to the Common Terminology Criteria for Adverse Events (CTCAE) version 5.0. Relevant data were extracted from medical records, hospitalization files and laboratory reports. Multiple irAEs were defined as the co-occurrence of two or more distinct immune-related adverse events. PFS was defined as the duration from the initiation of PD-1 inhibitor initiation to disease progression or all-cause death. OS was characterized as the time from treatment commencement to death from any cause. Tumor responses were assessed per Response Evaluation Criteria in Solid Tumors (RECIST) version 1.1, with contrast-enhanced computed tomography (CT) scans performed every 6–9 weeks for regular follow-up.

### 2.3. Statistical Analysis

Medians with interquartile ranges (IQRs) are used to express continuous variables, and comparisons were made using the Mann–Whitney U test. Categorical variables are presented as frequencies and percentages, with comparisons conducted using either the Pearson χ^2^ test or Fisher’s exact test, as deemed appropriate. For comparisons of TNM characteristics, the Fisher–Freeman–Halton exact test was used for cT, pT, and pN distributions, whereas Pearson’s chi-square test without continuity correction was used for cN, cM, and clinical stage-group distributions. The Kaplan–Meier technique was employed to produce PFS and OS curves, and the log-rank test was utilized to determine differences between the groups.

To avoid multicollinearity, ANA status and overall preexisting autoantibody status were entered into two separate multivariable Cox regression models rather than being included simultaneously. As PD-L1 data were unavailable for a considerable proportion of the overall cohort, a sensitivity analysis was additionally conducted for the 103 patients with available PD-L1 results. This analysis separately examined the prognostic value of PD-L1 expression, ANA status, and overall autoantibody status for PFS and OS.

The proportional hazards assumption was examined using Schoenfeld residuals. For variables that did not satisfy this assumption, Cox models with time-varying coefficients were fitted by introducing interactions between the corresponding covariates and log-transformed time. Univariable and multivariable Cox regression analyses were used to identify independent prognostic factors. Variables with a two-sided *p* value <0.10 in the univariable analysis were entered into the multivariable analysis to retain potentially relevant clinical confounders. Additional multivariable survival analyses were performed using the covariate structures reported in Supplementary Tables S4–S7, with treatment line, age, sex, and ECOG performance status included irrespective of their univariable statistical significance. Treatment line was categorized as first-line vs. second-line or later according to the recorded treatment line of the index PD-1 inhibitor-based regimen. These analyses included the overall cohort and patients with available PD-L1 results.

Statistical analyses were conducted using R software, version 4.5.1. All tests were two-sided, and *p* < 0.05 was considered statistically significant.

## 3. Results

### 3.1. Patient Characteristics

The baseline profiles of all 180 enrolled participants are summarized in Table 1 and Supplementary Table S1. The median age of the cohort was 67.0 years (IQR 61.0–73.0), and 85.6% were male. Most patients (92.8%) had an ECOG performance status of 0–1. Regarding PD-L1 expression, 43 patients (23.9%) had a CPS ≥10, 60 (33.3%) had a CPS < 10, and the remaining 77 (42.8%) had unknown PD-L1 status. The proportion of patients without available PD-L1 results reflected real-world testing practices at our institution during the study period. This missing information was addressed through a sensitivity analysis limited to patients with available PD-L1 data.

During treatment, 81 patients (45.0%) experienced irAEs of any grade. Baseline autoantibody positivity was detected in 95 patients (52.8%), including 66 patients (36.7% of the overall cohort) who were ANA-positive. Anti-Ro52 was the most commonly detected autoantibody, occurring in 24 patients (13.3%), whereas the positivity rate for each of the other individual autoantibodies was less than 5%.

### 3.2. Contributions of Preexisting ANA

The baseline characteristics stratified by ANA status are shown in Table 2 and Supplementary Table S2. No statistically significant intergroup differences were found in age, sex, ECOG performance status, histological differentiation, disease status, primary tumor site, hepatic metastasis, pulmonary metastasis, number of metastatic lesions, treatment line, or category of PD-1 inhibitors. Of note, among patients with valid PD-L1 measurements, ANA positivity was significantly associated with PD-L1 positivity (CPS ≥ 10) (53.5% vs. 33.3% in ANA-negative patients, *p* = 0.041).

Among patients without prior surgery (*n* = 118), the distributions of cT, cN, cM, and clinical stage group did not differ significantly according to pretreatment ANA status. Among patients with postoperative recurrence (*n* = 62), no statistically significant differences were observed in the pathological T and N categories at the time of the original surgery (all *p* > 0.05; Supplementary Table S8). The distributions of PD-1 inhibitors and chemotherapy regimens did not differ significantly between the ANA-negative and ANA-positive groups (*p* = 0.977 and *p* = 0.776, respectively; Supplementary Table S9).

### 3.3. Development of irAEs

**Figure 2:**
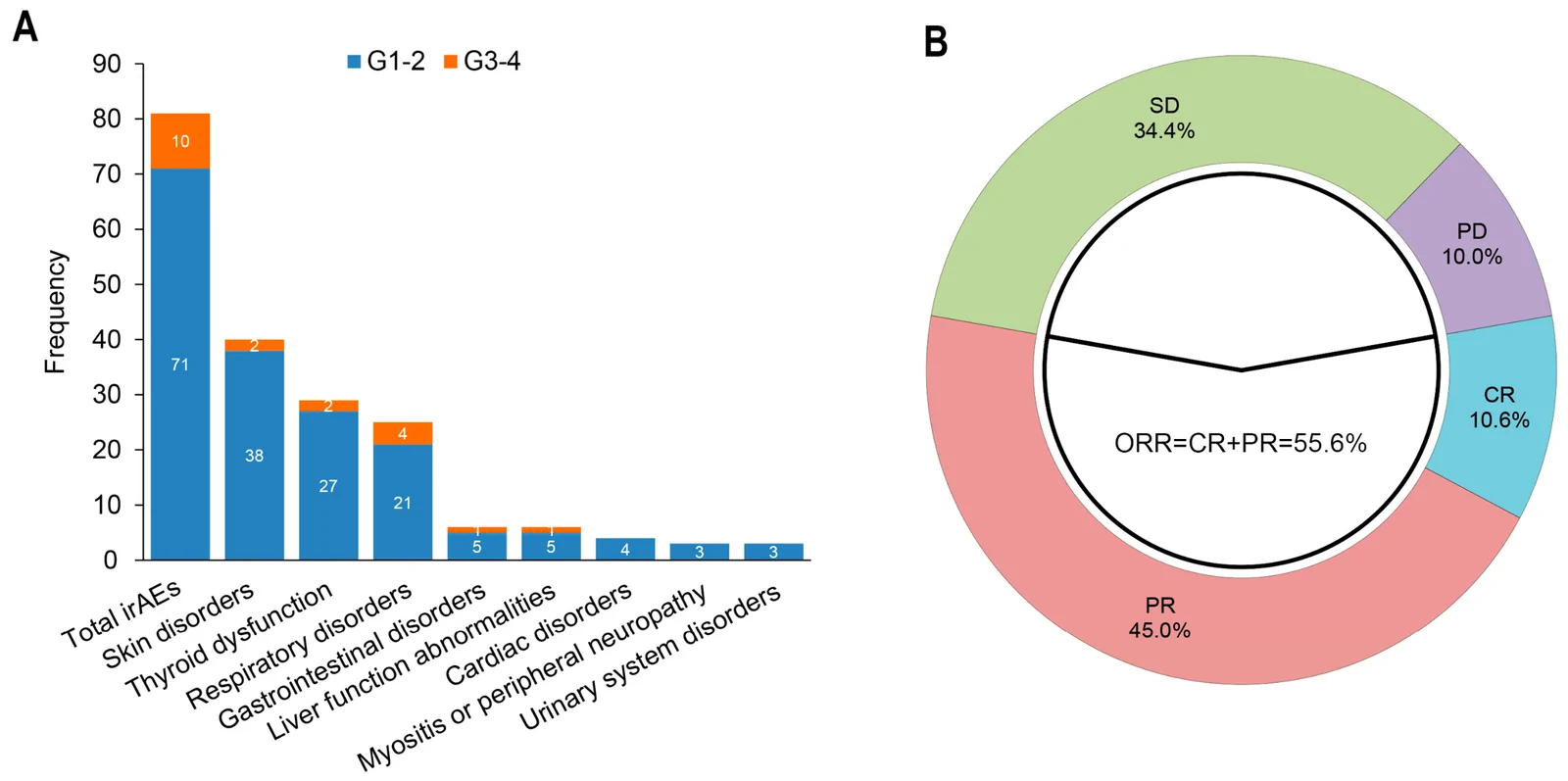
Toxicity and therapeutic response profiles. (**A**) Graded immune-related adverse events across different organ categories; (**B**) best overall response distribution. irAEs, immune-related adverse events; ESCC, esophageal squamous cell carcinoma; CR, complete response; PR, partial response; SD, stable disease; PD, progressive disease; ORR, objective response rate.

Figure 2A presents the frequency and distribution of irAEs. Among the 180 patients receiving anti-PD-1 therapy, 81 (45.00%) experienced irAEs of any grade, 10 (5.56%) experienced grade 3–4 events, and 30 (16.67%) developed multiple irAEs. Skin disorders were reported in 40 patients (22.22%), thyroid dysfunction in 29 (16.11%), and respiratory disorders in 25 (13.89%). Liver function abnormalities were reported in six patients (3.33%), while myositis or peripheral neuropathy was reported as a separate combined category in three patients (1.67%) (Supplementary Table S3).

As shown in Table 3, the overall incidence of irAEs was significantly higher in ANA-positive patients than in ANA-negative patients (60.61% vs. 35.96%, *p* = 0.001). A similar difference was observed for thyroid dysfunction, with a higher incidence in the ANA-positive group (28.79% vs. 8.77%, *p* < 0.001; Table 3). In contrast, anti-Ro52 positivity did not significantly correlate with the development of any-grade irAEs (Supplementary Table S3).

### 3.4. Evaluation of Efficacy

Table 4 summarizes the best overall response according to ANA status. In the entire cohort, CR was observed in 19 patients (10.56%), PR in 81 (45.00%), SD in 62 (34.44%), and PD in 18 (10.00%) (Figure 2B). The overall ORR was 55.56% (95% confidence interval [CI] 48.29–62.83%), while the DCR was 90.00% (95% CI 85.61–94.39%).

**Figure 3:**
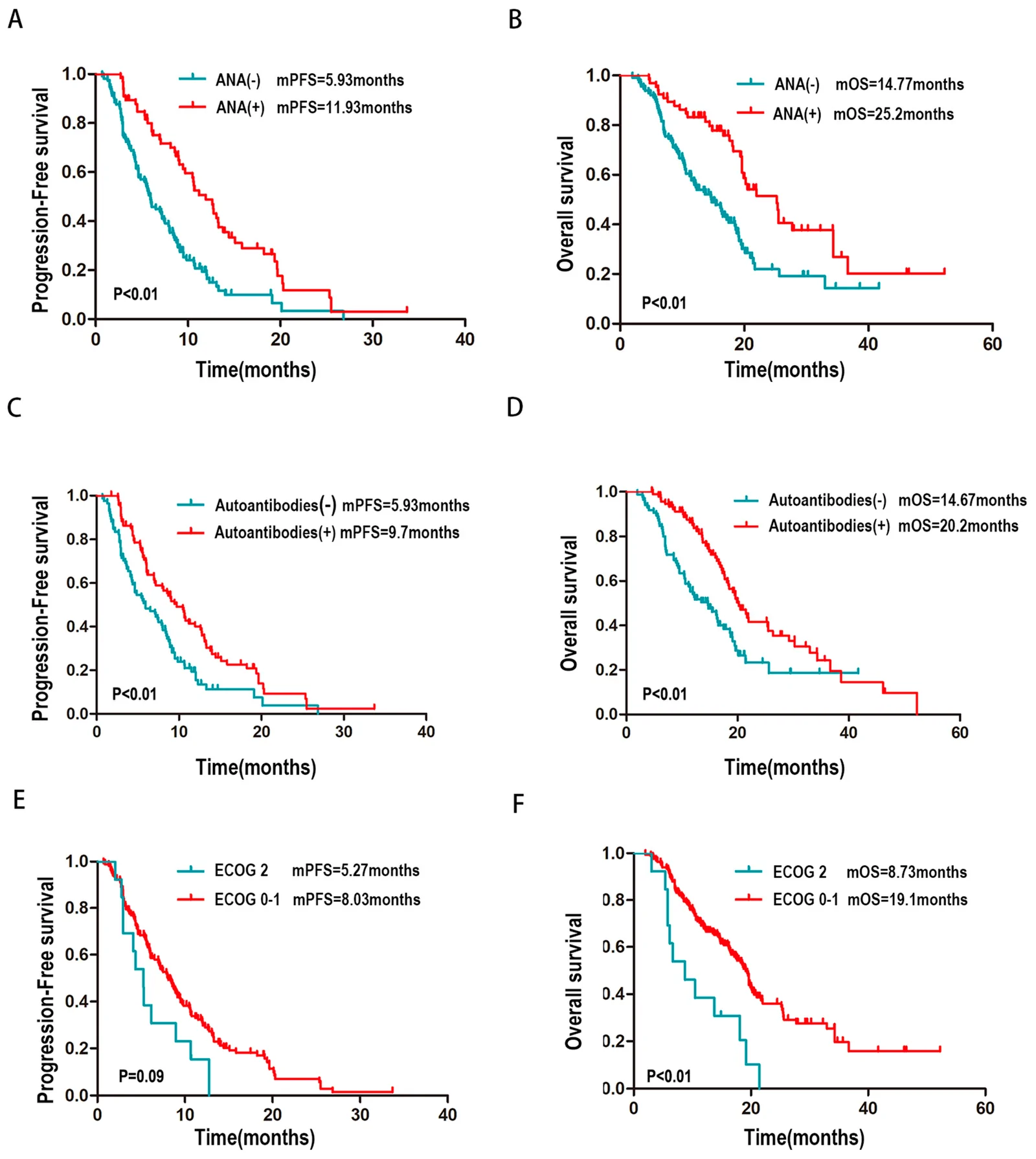
Kaplan–Meier curves for PFS and OS. (**A**) PFS by ANA status: ANA-positive (*n* = 66) vs. ANA-negative (*n* = 114), log-rank *p* < 0.01; (**B**) OS by ANA status: log-rank *p* < 0.01; (**C**) PFS by autoantibody status; (**D**) OS by autoantibody status; (**E**) PFS by ECOG PS; (**F**) OS by ECOG PS. Log-rank tests were applied for intergroup comparisons. PFS, progression-free survival; OS, overall survival; ANA: anti-nuclear antibody; ECOG, Eastern Cooperative Oncology Group; PS: performance status.

Treatment responses were subsequently compared between the ANA-positive and ANA-negative groups. Among ANA-positive patients, 10 (15.15%) achieved CR, 37 (56.06%) achieved PR, 18 (27.27%) had SD, and 1 (1.52%) had PD. Among ANA-negative patients, CR, PR, SD, and PD were recorded in 9 (7.89%), 44 (38.60%), 44 (38.60%), and 17 (14.91%) patients, respectively. The ORR was significantly higher in the ANA-positive group than in the ANA-negative group (71.21% vs. 46.49%, *p* = 0.002; Table 4). A similar difference was found for the DCR (98.48% vs. 85.09%, *p* = 0.009; Table 4). ANA-positive patients also had a significantly longer median PFS than ANA-negative patients (11.93 vs. 5.93 months, *p* < 0.01; Figure 3A). Median OS was likewise longer in the ANA-positive group (25.20 vs. 14.77 months, *p* < 0.01; Figure 3B).

### 3.5. Univariate and Multivariate Cox Analyses of PFS and OS

**Figure 4:**
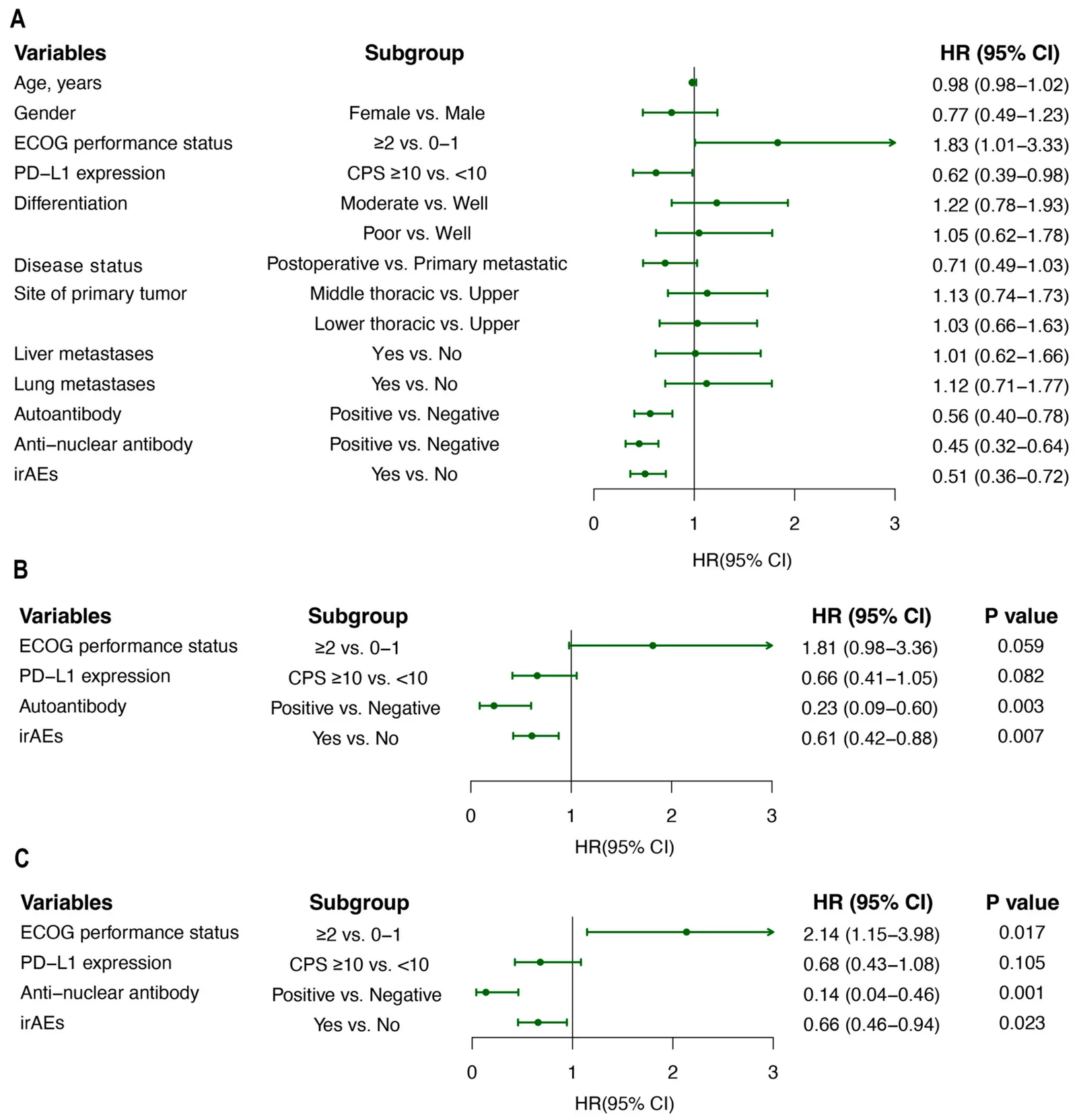
Univariate and multivariate Cox regression forest plots for PFS. (**A**) Univariate analysis; (**B**) multivariate model1 with autoantibodies; (**C**) multivariate model2 with ANA. PFS, progression-free survival; HR, hazard ratio; CI, confidence interval; ANA, anti-nuclear antibody.

Univariable Cox regression analysis of PFS identified ECOG performance status, PD-L1 CPS, baseline autoantibody positivity, ANA positivity, and the occurrence of irAEs as significant favorable prognostic factors (Supplementary Table S4 and Figure 4A). In the respective multivariable time-varying Cox models, favorable ECOG performance status, autoantibody positivity (HR = 0.23, 95% CI 0.09–0.60; *p* = 0.003), ANA positivity (HR = 0.14, 95% CI 0.04–0.46; *p* = 0.001), and the occurrence of irAEs remained independently associated with longer PFS (Supplementary Table S4; Figure 4B,C).

As PD-L1 data were unavailable for a substantial proportion of the overall cohort, we conducted a sensitivity analysis among the 103 patients with available PD-L1 results (Supplementary Tables S6 and S7). In the respective multivariable models, ECOG performance status and baseline ANA positivity remained associated with both PFS and OS, whereas overall autoantibody positivity was associated with PFS only. PD-L1 expression was not significantly associated with either endpoint. Time-varying effects of ANA and overall autoantibody status were observed in the corresponding Cox models. Associations between ANA positivity and survival outcomes were observed in both the overall cohort and the sensitivity cohort. The ANA main effect and its interaction with time were jointly associated with PFS and OS in the overall cohort (both joint *p* < 0.001). At 180 days, the adjusted HRs for ANA-positive versus ANA-negative patients were 0.461 (95% CI 0.299–0.709) for PFS and 0.284 (95% CI 0.131–0.613) for OS. In patients with available PD-L1 results, the corresponding joint *p* values were 0.001 for PFS and 0.011 for OS. These are time-varying associations and do not imply statistically significant effects throughout follow-up. The PFS findings remain exploratory because of uncertainty regarding the recorded censoring of 16 patients.

**Figure 5:**
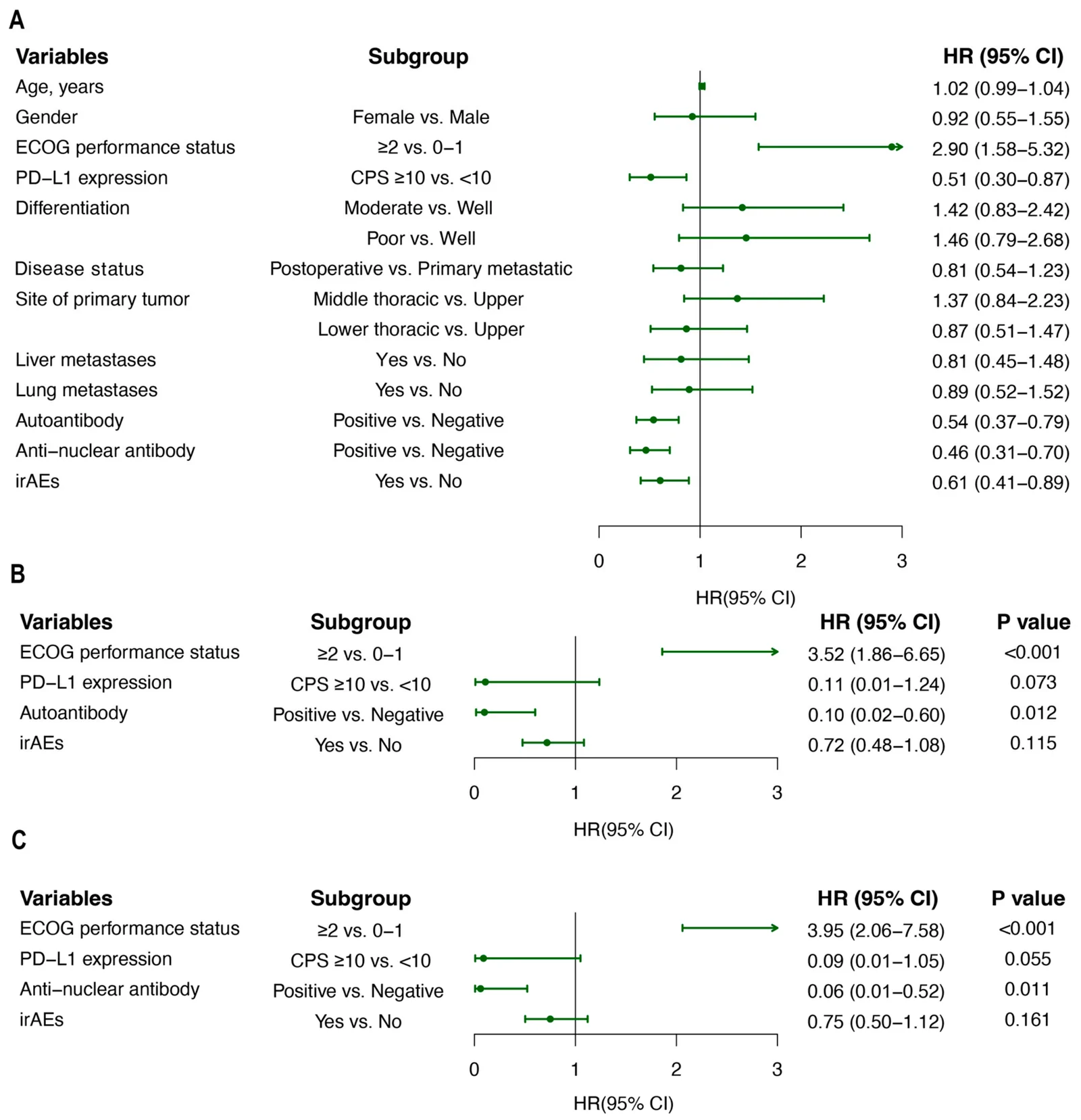
Univariate and multivariate Cox regression forest plots for OS. (**A**) Univariate analysis; (**B**) multivariate model1 with autoantibodies; (**C**) multivariate model2 with ANA. OS, overall survival; HR, hazard ratio; CI, confidence interval; ANA, anti-nuclear antibody.

For OS, the univariable Cox analysis showed that ECOG performance status, PD-L1 CPS, baseline autoantibody positivity, ANA positivity, and irAEs had significant prognostic effects (Supplementary Table S5 and Figure 5A). The corresponding multivariable analyses indicated that favorable ECOG performance status, autoantibody positivity (HR = 0.10, 95% CI 0.02–0.60; *p* = 0.012), and improved OS were independently and strongly associated with ANA positivity (HR = 0.06, 95% CI 0.01–0.52; *p* = 0.011). Neither PD-L1 CPS nor the occurrence of irAEs was significantly associated with OS in the corresponding multivariable models (Supplementary Table S5; Figure 5B,C).

## 4. Discussion

PD-1 inhibitor-based regimens have become an important component of systemic therapy for advanced ESCC [16,17,18]. However, substantial variation in treatment response and the unpredictable development of irAEs remain important challenges in clinical practice [19]. PD-L1 expression is widely used to inform treatment selection [20], but its assessment usually requires tumor tissue and provides limited information regarding irAE risk. Easily accessible blood-based biomarkers that may help evaluate both treatment outcomes and toxicity are therefore of clinical interest [21,22]. In this study, pretreatment ANA positivity was associated with higher response rates, longer survival, and a greater incidence of irAEs among patients with ESCC receiving PD-1 inhibitor-based therapy. These findings suggest that ANA may have potential as an adjunctive host-related biomarker, although further validation is required.

Although pre-existing ANA has been detected across 17–51% of patients with various malignancies, its role in regulating tumor immunity and ICI treatment outcomes remains controversial [23]. Some studies have associated ANA positivity with increased tumor immunogenicity and improved long-term outcomes [24,25], whereas others have found no survival advantage or have observed greater toxicity without improved treatment response [26]. Differences in tumor type, ANA titer threshold, treatment setting, and tumor immune microenvironment may contribute to these inconsistent results. As ESCC is characterized by relatively high immunogenicity and substantial immune-cell infiltration, host immune status may influence the activity of ICIs in this disease [27]. Nevertheless, evidence regarding the clinical relevance of pretreatment ANA in ICI-treated ESCC remains limited.

In the present cohort, ANA-positive patients had higher ORR and DCR and longer median PFS and OS than ANA-negative patients. ANA positivity also remained associated with survival outcomes in the adjusted Cox analyses. In addition, ANA positivity was more frequent among patients with CPS ≥10. One possible explanation is that systemic immune activation and an inflamed tumor microenvironment may share related immunological features, including increased PD-L1 expression. Associations between circulating autoantibodies and tumor immune phenotypes have also been reported in other malignancies [28]. However, the cross-sectional association observed in this study cannot establish a causal relationship between ANA and PD-L1 expression.

The favorable outcomes observed in ANA-positive patients may be related to differences in baseline host immunity. ANA positivity may reflect a state of immune activation that could facilitate antitumor immune responses following PD-1 blockade [24,29]. The same immune tendency might also increase susceptibility to autoreactivity and irAEs after immune checkpoint inhibition [30]. These mechanisms remain hypothetical, and the present observational data cannot determine whether ANA directly contributes to treatment response or merely reflects other features of the host immune environment [31].

ANA-positive patients experienced a higher overall incidence of irAEs, with the clearest difference observed for thyroid dysfunction. A numerical increase in skin toxicities was also observed in the ANA-positive group, although the difference was not statistically significant. These findings suggest that pretreatment autoimmune predisposition may be related to susceptibility to irAEs, particularly endocrine toxicity and thyroid dysfunction [32]. If confirmed in prospective studies, ANA status might help identify patients who could benefit from closer monitoring of thyroid function [33,34]. However, ANA positivity alone should not be regarded as sufficient evidence for predicting whether an individual patient will develop an irAE.

As PD-L1 results were unavailable for a substantial proportion of the overall cohort, a sensitivity analysis was conducted among the 103 patients with complete PD-L1 data. ANA remained associated with survival outcomes after adjustment for PD-L1 and other clinical variables in this subgroup, whereas PD-L1 expression was not independently associated with PFS or OS. Separate models were fitted for ANA and overall autoantibody status to reduce collinearity. The consistency between the full-cohort and sensitivity analyses suggests that the observed association with ANA may not be explained entirely by PD-L1 expression. Nevertheless, the reduced sample size and complete-case design limit the strength of this conclusion.

Several limitations should be considered. First, PD-L1 results were missing for 42.78% of patients because testing was not routinely performed during the early study period. The possibility of selection bias and residual confounding by PD-L1 therefore cannot be excluded [35]. Second, the retrospective, single-center design and moderate sample size restrict the generalizability of the findings. Third, treatment duration was not incorporated into the regression models, although longer exposure may increase the opportunity for irAEs to occur [36]. Reliable patient-level counts of the PD-1 inhibitor and chemotherapy cycles actually administered were also unavailable, preventing a detailed comparison of cumulative treatment exposure between the ANA groups. Fourth, irAEs were treated as baseline covariates in the Cox analyses despite occurring during follow-up. This approach may introduce immortal time bias; consequently, the observed relationship between irAEs and survival should be regarded as exploratory. Fifth, changes in ANA titers during treatment were not evaluated [26], and analyses according to ANA immunofluorescence patterns were not performed. Therefore, we could not determine whether the observed associations with treatment response, survival, or irAEs varied by ANA pattern. Finally, all patients received PD-1 inhibitor-based immunochemotherapy, and no chemotherapy-only or other non-ICI comparator group was included. Therefore, the observed associations cannot distinguish general prognostic associations from treatment-specific predictive effects or establish that ANA identifies patients who derive greater benefit from adding PD-1 blockade. Future comparative studies with an appropriate non-ICI group and evaluation of the treatment-by-ANA interaction are needed to address this question. An additional limitation concerns PFS ascertainment. Sixteen patients were recorded as censored for PFS before a subsequently documented death during OS follow-up. The clinical basis for these earlier censoring dates could not be established from the available dataset. The recorded PFS times and event indicators were retained in the additional analyses; therefore, the PFS findings should be interpreted as exploratory and potentially affected by misclassification or informative censoring. Adjustment for treatment line does not resolve this uncertainty.

In conclusion, pretreatment ANA positivity was associated with higher response rates, longer survival, and an increased incidence of irAEs—particularly thyroid dysfunction—in this cohort of patients with ESCC receiving PD-1 inhibitor-based immunochemotherapy. These hypothesis-generating findings require confirmation in larger, multicenter prospective studies with standardized PD-L1 testing, time-dependent assessment of irAEs, and an appropriate non-ICI comparison group. The present study does not establish clinical predictive utility, and further validation is required before ANA can be used to guide treatment selection or individual toxicity risk assessment.

## Figures and Tables

**Table 1:** Baseline characteristics of the overall ESCC cohort (*N* = 180).

Characteristic	Value
Age, years, median (IQR)	67.0 (61.0–73.0)
Gender, male, *n* (%)	154 (85.6)
ECOG performance status, *n* (%)	
0–1	167 (92.8)
≥2	13 (7.2)
PD-L1 expression, *n* (%)	
CPS < 10	60 (33.3)
CPS ≥ 10	43 (23.9)
Unknown	77 (42.8)
Tumor differentiation, *n* (%)	
Well	30 (16.7)
Moderate	105 (58.3)
Poor	45 (25.0)
Patients without prior surgery, *n* (%)	118 (65.6)
Liver metastases, *n* (%)	24 (13.3)
Lung metastases, *n* (%)	28 (15.6)
Any autoantibody positivity, *n* (%)	95 (52.8)
ANA-positive, *n* (%)	66 (36.7)
Anti-Ro52-positive, *n* (%)	24 (13.3)
First-line PD-1 inhibitor, *n* (%)	165 (91.7)
Any-grade irAEs, *n* (%)	81 (45.0)
PD-1 inhibitor, *n* (%)	
Camrelizumab	64 (35.6)
Tislelizumab	28 (15.6)
Toripalimab	53 (29.4)
Sintilimab	35 (19.4)
Chemotherapy regimen, *n* (%)	
Nab-paclitaxel + carboplatin	82 (45.6)
Nab-paclitaxel + lobaplatin	42 (23.3)
Nab-paclitaxel + nedaplatin	38 (21.1)
Nab-paclitaxel + cisplatin	18 (10.0)

Abbreviations: ESCC, esophageal squamous cell carcinoma; IQR, interquartile range; ECOG, Eastern Cooperative Oncology Group; PD-L1, programmed death-ligand 1; CPS, combined positive score; ANA, antinuclear antibody; irAEs, immune-related adverse events.

**Table 2:** Baseline characteristics stratified by pretreatment ANA status.

Characteristic	ANA-Negative (*N* = 114)	ANA-Positive (*N* = 66)	*p* Value
Age, years, median (IQR)	68.0 (62.3–74.0)	66.5 (60.0–71.8)	0.180
Male gender, *n* (%)	98 (86.0)	56 (84.9)	0.837
ECOG performance status ≥ 2, *n* (%)	7 (6.1)	6 (9.1)	0.553
PD-L1 CPS ≥ 10 (among known), *n*/*N* (%) *	20/60 (33.3)	23/43 (53.5)	0.041
Autoantibody-positive, *n* (%)	29 (25.4)	66 (100.0)	<0.001
Anti-Ro52-positive, *n* (%)	10 (8.8)	14 (21.2)	0.018
Liver metastases, *n* (%)	14 (12.3)	10 (15.2)	0.585
Lung metastases, *n* (%)	16 (14.0)	12 (18.2)	0.459
First-line PD-1 inhibitor, *n* (%)	105 (92.1)	60 (90.9)	0.780
Any-grade irAEs, *n* (%)	41 (36.0)	40 (60.6)	0.001

* Among patients with known PD-L1 status (total 103).

**Table 3:** Selected irAEs stratified by autoantibody and ANA status.

irAEs, *n* (%)	Autoantibody-Negative (*n* = 85)	Autoantibody-Positive (*n* = 95)	*p* Value	ANA-Negative (*n* = 114)	ANA-Positive (*n* = 66)	*p* Value
Any-grade irAEs	41 (48.24)	40 (42.11)	0.414	41 (35.96)	40 (60.61)	0.001
Skin disorders	17 (20.00)	23 (24.21)	0.498	22 (19.30)	18 (27.27)	0.215
Thyroid dysfunction	8 (9.41)	21 (22.11)	0.021	10 (8.77)	19 (28.79)	<0.001
Respiratory disorders	8 (9.41)	17 (17.89)	0.100	13 (11.40)	12 (18.18)	0.205
Myositis or peripheral neuropathy	0 (0)	3 (3.16)	0.248	0 (0)	3 (4.55)	0.048

Only adverse events with notable differences or clinical relevance are shown. *p* values: Pearson’s chi-squared or Fisher’s exact test. Complete adverse event data including anti-Ro52 are provided in Supplementary Table S3.

**Table 4:** Summary of tumor responses according to pretreatment ANA status.

Best Overall Response	Total (*N* = 180)	ANA-Negative (*n* = 114)	ANA-Positive (*n* = 66)	*p* Value
Complete response, *n* (%)	19 (10.56)	9 (7.89)	10 (15.15)	–
Partial response, *n* (%)	81 (45.00)	44 (38.60)	37 (56.06)	–
Stable disease, *n* (%)	62 (34.44)	44 (38.60)	18 (27.27)	–
Progressive disease, *n* (%)	18 (10.00)	17 (14.91)	1 (1.52)	–
ORR, *n* (%) (95%CI)	100 (55.56) (48.29–62.83)	53 (46.49) (37.34–55.92)	47 (71.21) (60.30–82.12)	0.002
DCR, *n* (%) (95%CI)	162 (90.00) (85.61–94.39)	97 (85.09) (77.55–90.53)	65 (98.48) (91.74–99.97)	0.009

*p* values from Pearson’s chi-square test; “–” indicates not calculated for individual response categories. Abbreviations: CR, complete response; PR, partial response; SD, stable disease; PD, progressive disease; ORR, objective response rate; DCR, disease control rate; CI, confidence interval.

## Data Availability

The datasets generated and analyzed during this study are not publicly available due to institutional data usage restrictions. Data can be obtained from the corresponding author upon reasonable request and approval from Nanjing Drum Tower Hospital.

## Supplementary Materials

The following supporting information can be downloaded at: https://www.mdpi.com/article/10.3390/curroncol33100596/s1, Table S1: Demographics and baseline characteristics (full dataset); Table S2: Comparison of demographic and baseline characteristics according to ANA status (full dataset); Table S3: irAEs stratified by autoantibody, anti-Ro52 antibody, and ANA status (full dataset); Table S4: Univariable Cox proportional hazards regression and multivariable time-varying coefficient Cox regression analyses for PFS (overall cohort); Table S5: Univariable Cox proportional hazards regression and multivariable time-varying coefficient Cox regression analyses for OS (overall cohort); Table S6: Univariable Cox proportional hazards regression and multivariable time-varying coefficient Cox regression analyses for PFS (sensitivity analysis restricted to patients with known PD-L1 expression); Table S7: Univariable Cox proportional hazards regression and multivariable time-varying coefficient Cox regression analyses for OS (sensitivity analysis restricted to patients with known PD-L1 expression); Table S8: TNM characteristics according to pretreatment ANA status; Table S9: Treatment characteristics according to pretreatment ANA status.
