# Supplementary material for: The Role of Antinuclear Antibody in Predicting the Efficacy and Immune-Related Adverse Events of Immunochemotherapy in Esophageal Squamous Cell Carcinoma: A Real-World Analysis
Source: Curr Oncol. 2026 Oct 1;33(10):596. doi: 10.3390/curroncol33100596 (PMC13703202; doi:10.3390/curroncol33100596)
Supplement: Supplementary file 1 [file curroncol-33-00596-s001.zip › curroncol-4532698-supplementary.pdf]

Supplementary Table S1. Demographics and baseline characteristics (Full Dataset)

Variable	Patients (N=180)
Age, years, median (IQR)	67.00 (61.00, 73.00)
Gender, n (%)	
Female	26 (14.44)
Male	154 (85.56)
ECOG performance status, n (%)	
0-1	167 (92.78)
≥2	13 (7.22)
PD-L1 expression, n (%)	
CPS <10	60 (33.33)
CPS ≥10	43 (23.89)
Unknown	77 (42.78)
Differentiation, n (%)	
Well differentiated	30 (16.67)
Moderately differentiated	105 (58.33)
Poorly differentiated	45 (25.00)
Cancer type, n (%)	
Primary metastatic	118 (65.56)
Postoperative recurrence	62 (34.44)
Site of primary tumor, n (%)	
Upper thoracic	36 (20.00)
Middle thoracic	80 (44.44)
Lower thoracic	64 (35.56)
Liver metastases, n (%)	24 (13.33)
Lung metastases, n (%)	28 (15.56)
Number of metastatic sites, n (%)	
< 2	141 (78.33)
≥2	39 (21.67)
Autoantibody, n (%)	
Positive	95 (52.78)
Negative	85 (47.22)
ANA, n (%)	
Positive	66 (36.67)
Negative	114 (63.33)
Anti-Ro52 antibody, n (%)	
Positive	24 (13.33)
Negative	156 (86.67)
Lines of PD-1 Inhibitor therapy, n (%)	

1	165 (91.67)
⩾2	15 (8.33)
PD-1 Inhibitors, n (%)	
Camrelizumab	64 (35.56)
Tislelizumab	28 (15.56)
Toripalimab	53 (29.44)
Sintilimab	35 (19.44)
irAE, n (%)	81 (45.00)

Abbreviations: IQR, interquartile range; ECOG, Eastern Cooperative Oncology Group; PD-L1, programmed death-ligand 1; CPS, combined positive score; PD-1, programmed death-1; irAEs, immune-related adverse events; ANA, anti-nuclear antibody.

Supplementary Table S2. Comparison of demographic and baseline characteristics according to ANA status (Full Dataset)

Variable	Total (N=180)	ANA negative (n=114)	ANA positive (n=66)	*P* value
Age, years, median (IQR)	67.00 (61.00, 73.00)	68.00 (62.25, 74.00)	66.50 (60.00, 71.75)	0.180[$]
Gender, n (%)				0.837[*]
Female	26 (14.44)	16 (14.04)	10 (15.15)	
Male	154 (85.56)	98 (85.96)	56 (84.85)	
ECOG performance status, n (%)				0.553[#]
0-1	167 (92.78)	107 (93.86)	60 (90.91)	
≥2	13 (7.22)	7 (6.14)	6 (9.09)	
PD-L1 expression, n (%) [†]				0.041[*]
CPS <10	60 (58.25)	40 (66.67)	20 (46.51)	
CPS ≥10	43 (41.75)	20 (33.33)	23 (53.49)	
Differentiation, n (%)				0.116[*]
Well differentiated	30 (16.67)	14 (12.28)	16 (24.24)	
Moderately differentiated	105 (58.33)	70 (61.40)	35 (53.03)	
Poorly differentiated	45 (25.00)	30 (26.32)	15 (22.73)	
Disease status, n (%)				0.680[*]
Unresectable advanced	118 (65.56)	76 (66.67)	42 (63.64)	
Postoperative recurrence	62 (34.44)	38 (33.33)	24 (36.36)	
Site of primary tumor, n (%)				0.115[*]
Upper thoracic	36 (20.00)	19 (16.67)	17 (25.76)	
Middle thoracic	80 (44.44)	57 (50.00)	23 (34.85)	
Lower thoracic	64 (35.56)	38 (33.33)	26 (39.39)	
Liver metastases, n (%)				0.585[*]
No	156 (86.67)	100 (87.72)	56 (84.85)	
Yes	24 (13.33)	14 (12.28)	10 (15.15)	

Lung metastases, n (%)				0.459*
No	152 (84.44)	98 (85.96)	54 (81.82)	
Yes	28 (15.56)	16 (14.04)	12 (18.18)	
Number of metastatic sites, n (%)				0.793*
< 2	141 (78.33)	90 (78.95)	51 (77.27)	
≥2	39 (21.67)	24 (21.05)	15 (22.73)	
Autoantibody, n (%)				＜0.001#
Positive	95 (52.78)	29 (25.44)	66 (100.0)	
Negative	85 (47.22)	85 (74.56)	0 (0.0)	
Anti-Ro52 antibody, n (%)				0.018*
Positive	24 (13.33)	10 (8.77)	14 (21.21)	
Negative	156 (86.67)	104 (91.23)	52 (78.79)	
Lines of PD-1 Inhibitor therapy, n (%)				0.780*
1	165 (91.67)	105 (92.11)	60 (90.91)	
⩾2	15 (8.33)	9 (7.89)	6 (9.09)	
PD-1 Inhibitors, n (%)				0.977*
Camrelizumab	64 (35.56)	40 (35.09)	24 (36.36)	
Tislelizumab	28 (15.56)	17 (14.91)	11 (16.67)	
Toripalimab	53 (29.44)	34 (29.82)	19 (28.79)	
Sintilimab	35 (19.44)	23 (20.18)	12 (18.18)	
irAEs, n (%)				0.001†
No	99 (55.00)	73 (64.04)	26 (39.39)	
Yes	81 (45.00)	41 (35.96)	40 (60.61)	

Abbreviations: IQR, inter-quartile range; ECOG, Eastern Cooperative Oncology Group; PD-L1, programmed death ligand 1; CPS, combined positive score; PD-1, programmed death receptor 1; irAEs, immune-related adverse events; ANA, anti-nuclear antibody.

*Pearson's chi-squared test. #Fisher's exact test. † Percentages for PD-L1 expression are based on patients with known PD-L1 status (n=103). $ Mann–Whitney U-test.

Supplementary Table S3. irAEs stratified by autoantibody, Anti-Ro52 antibody, and ANA status (Full Dataset)

Adverse Event, n (%)	Total N=180	Autoantibody			Anti-Ro52 antibody			ANA		
		Negative n=85	Positive n=95	*P* value	Negative n=156	Positive n=24	*P* value	Negative n=114	Positive n=66	*P* value
Skin disorders	40 (22.22)	17 (20.00)	23 (24.21)	0.498*	36 (23.08)	4 (16.67)	0.482*	22 (19.30)	18 (27.27)	0.215*
Thyroid dysfunction	29 (16.11)	8 (9.41)	21 (22.11)	0.021*	27 (17.31)	2 (8.33)	0.377#	10 (8.77)	19 (28.79)	<0.001*
Cardiac disorders	4 (2.22)	1 (1.18)	3 (3.16)	0.623#	3 (1.92)	1 (4.17)	0.439#	2 (1.75)	2 (3.03)	0.625#
Respiratory disorders	25 (13.89)	8 (9.41)	17 (17.89)	0.100*	23 (14.74)	2 (8.33)	0.537#	13 (11.40)	12 (18.18)	0.205*
Gastrointestinal disorders	6 (3.33)	3 (3.53)	3 (3.16)	1.000#	6 (3.85)	0 (0)	1.000#	3 (2.63)	3 (4.55)	0.670#
Liver function abnormalities	6 (3.33)	3 (3.53)	3 (3.16)	1.000#	5 (3.21)	1 (4.17)	0.582#	3 (2.63)	3 (4.55)	0.670#
Myositis or peripheral neuropathy	3 (1.67)	0 (0)	3 (3.16)	0.248#	2 (1.28)	1 (4.17)	0.351#	0 (0)	3 (4.55)	0.048#
Urinary system disorders	3 (1.67)	1 (1.18)	2 (2.11)	1.000#	3 (1.92)	0 (0)	1.000#	2 (1.75)	1 (1.52)	1.000#

Abbreviations: irAEs, immune-related adverse events; ANA, anti-nuclear antibody. *Pearson's chi-squared test. #Fisher's exact test.

Supplementary Table S4. Univariable Cox proportional hazards regression and multivariable time-varying coefficient Cox regression analyses for PFS (overall cohort)

Variables	Univariable HR (95% CI)	Univariable *P* value	Model 1 #		Model 2 $	
			Multivariable HR (95% CI)	Multivariable *P* value	Multivariable HR (95% CI)	Multivariable *P* value
Age, years	0.98 (0.98- 1.02)	0.833				
Gender (Female vs. Male)	0.77 (0.49-1.23)	0.279				
ECOG performance status (≥2 vs. 0-1)	1.83 (1.01-3.33)	0.047	1.81 (0.98-3.36)	0.059	2.14 (1.15-3.98	0.017
PD-L1 expression (CPS ≥10 vs. <10)						
CPS ≥10 vs. <10	0.62 (0.39-0.98)	0.042	0.66 (0.41-1.05)	0.082	0.68 (0.43-1.08)	0.105
Unknown vs. <10	1.13 (0.78-1.64)	0.517	1.07 (0.73-1.56)	0.744	1.03 (0.70-1.50)	0.889
Differentiation						
Moderate vs. Well	1.22 (0.78-1.93)	0.386				
Poor vs. Well	1.05 (0.62-1.78)	0.861				
Cancer type (Postoperative recurrence vs. Primary metastatic)	0.71 (0.49-1.03)	0.070				
Site of primary tumor						
Middle thoracic vs. Upper thoracic	1.13 (0.74-1.73)	0.575				
Lower thoracic vs. Upper thoracic	1.03 (0.66-1.63)	0.890				
Liver metastases (Yes vs. No)	1.01 (0.62-1.66)	0.966				
Lung metastases (Yes vs. No)	1.12 (0.71-1.77)	0.619				
Number of metastatic sites (≥2 vs. <2)	1.04 (0.69-1.56)	0.858				

Autoantibody (Positive vs. Negative) †	0.56 (0.40-0.78)	0.001	0.23 (0.09-0.60)	0.003		
Anti-nuclear antibody (Positive vs. Negative) *, †	0.45 (0.32-0.64)	<0.001			0.14 (0.04-0.46)	0.001
Anti-Ro52 antibody (Positive vs. Negative)	0.99 (0.60-1.62)	0.965				
Lines of PD-1 Inhibitor therapy (⩾2 vs. 1)	1.61 (0.92-2.81)	0.093				
PD-1 Inhibitors						
Tislelizumab vs. Camrelizumab	0.95 (0.57-1.59)	0.856				
Toripalimab vs. Camrelizumab	1.23 (0.82-1.85)	0.314				
Sintilimab vs. Camrelizumab	1.55 (0.97-2.48)	0.068				
irAE (Yes vs. No)	0.51 (0.36-0.72)	<0.001	0.61 (0.42-0.88)	0.007	0.66 (0.46-0.94)	0.023

Abbreviations: PFS, Progression-Free Survival; HR, hazard ratio; 95% CI, 95% confidence interval; ECOG, Eastern Cooperative Oncology Group; PD-L1, programmed death ligand 1; PD-1, programmed death receptor 1; irAEs, immune-related adverse events; ANA, anti-nuclear antibody.

* Autoantibody and ANA were included separately in the multivariate analysis to avoid multicollinearity.

† In the multivariable time-varying coefficient Cox regression, significant time-varying effects were observed for autoantibody in Model 1 (interaction HR=1.816, 95% CI: 1.090–3.027, *P*=0.022) and anti-nuclear antibody in Model 2 (interaction HR=2.009, 95% CI: 1.112–3.632, *P*=0.021).

# Model 1 was adjusted for ECOG performance status, PD-L1 expression, autoantibody, and irAEs, with an autoantibody-by-log(time) interaction term included to account for non-proportional hazards.

$ Model 2 was adjusted for ECOG performance status, PD-L1 expression, ANA, and irAEs, with an ANA-by-log(time) interaction term included to account for non-proportional hazards. Separate multivariate models were constructed for ANA and autoantibody to avoid multicollinearity. Time-varying coefficient Cox regression with covariate-by-log(time) interaction was used when the proportional hazards assumption was violated.

Supplementary Table S5. Univariable Cox proportional hazards regression and multivariable time-varying coefficient Cox regression analyses for OS (overall cohort)

Variables	Univariable HR (95% CI)	Univariable *P* value	Model 1 #		Model 2 $	
			Multivariable HR (95% CI)	Multivariable *P* value	Multivariable HR (95% CI)	Multivariable *P* value
Age, years	1.02 (0.99-1.04)	0.143				
Gender (Female vs. Male)	0.92 (0.55-1.55)	0.761				

ECOG performance status (≥2 vs. 0-1)	2.90 (1.58-5.32)	0.001	3.52 (1.86-6.65)	<0.001	3.95 (2.06-7.58)	<0.001
PD-L1 expression †						
CPS ≥10 vs. <10	0.51 (0.30-0.87)	0.012	0.11 (0.01- 1.24)	0.073	0.09 (0.01-1.05)	0.055
Unknown vs. <10	1.05 (0.69-1.59)	0.835	0.30 (0.06-1.65)	0.167	0.24 (0.05-1.32)	0.101
Differentiation						
Moderate vs. Well	1.42 (0.83-2.42)	0.201				
Poor vs. Well	1.46 (0.79-2.68)	0.227				
Cancer type (Postoperative recurrence vs. Primary metastatic)	0.81 (0.54-1.23)	0.322				
Site of primary tumor						
Middle thoracic vs. Upper	1.37 (0.84-2.23)	0.205				
Lower thoracic vs. Upper	0.87 (0.51-1.47)	0.589				
Liver metastases (Yes vs. No)	0.81 (0.45-1.48)	0.498				
Lung metastases (Yes vs. No)	0.89 (0.52-1.52)	0.674				
Number of metastatic sites (≥2 vs. <2)	0.86 (0.54-1.39)	0.540				
Autoantibody (Positive vs. Negative) †	0.54 (0.37-0.79)	0.001	0.10 (0.02- 0.60)	0.012		
Anti-nuclear antibody (Positive vs. Negative) *, †	0.46 (0.31-0.70)	<0.001			0.06 (0.01-0.52)	0.011
Anti-Ro52 antibody (Positive vs. Negative)	0.97 (0.56-1.68)	0.917				
Lines of PD-1 Inhibitor therapy (≥2 vs. 1)	1.29 (0.71-2.35)	0.404				
PD-1 Inhibitors						
Tislelizumab vs. Camrelizumab	1.07 (0.60-1.92)	0.814				
Toripalimab vs. Camrelizumab	1.51 (0.95-2.40)	0.081				
Sintilimab vs. Camrelizumab	1.64 (0.95-2.83)	0.078				

irAEs (Yes vs. No)	0.61 (0.41-0.89)	0.010	0.72 (0.476-1.084)	0.115	0.75 (0.50-1.12)	0.161

Abbreviations: OS, Overall Survival; HR, hazard ratio; 95% CI, 95% confidence interval; ECOG, Eastern Cooperative Oncology Group; PD-L1, programmed death ligand 1; PD-1, programmed death receptor 1; irAEs, immune-related adverse events; ANA, anti-nuclear antibody.

[*] Autoantibody and anti-nuclear antibody were included separately in the multivariate analysis to avoid multicollinearity.

[†] In the multivariable time-varying coefficient Cox regression, significant time-varying effects were observed for autoantibody positivity in Model 1 (interaction HR = 2.142, 95% CI: 1.026–4.475, *P* = 0.043) and anti-nuclear antibody positivity in Model 2 (interaction HR = 2.329, 95% CI: 1.021–5.310, *P* = 0.044). No significant time-varying effects were observed for PD-L1 CPS ≥10 or unknown PD-L1 status, using PD-L1 CPS <10 as the reference.

[#] Model 1 included ECOG performance status, PD-L1 expression, autoantibody status, and irAEs, with covariate-by-log(time) interaction terms for PD-L1 expression and autoantibody status to account for non-proportional hazards.

[$] Model 2 included ECOG performance status, PD-L1 expression, ANA status, and irAEs, with covariate-by-log(time) interaction terms for anti-nuclear antibody status and PD-L1 expression to account for non-proportional hazards. Separate multivariate models were constructed for ANA and autoantibody to avoid multicollinearity. Time-varying coefficient Cox regression with covariate-by-log(time) interaction was used when the proportional hazards assumption was violated.

Supplementary Table S6. Univariable Cox proportional hazards regression and multivariable time-varying coefficient Cox regression analyses for PFS (sensitivity analysis restricted to patients with known PD-L1 expression)

Variables	Univariable HR (95% CI)	Univariable *P* value	Model 1 [#]		Model 2 [$]	
			Multivariable HR (95% CI)	Multivariable *P* value	Multivariable HR (95% CI)	Multivariable *P* value
Age, years	1.00 (0.97- 1.03)	0.968				
Gender (Female vs. Male)	0.70 (0.36-1.37)	0.297				
ECOG performance status (≥2 vs. 0-1)	2.23 (1.13-4.40)	0.021	2.40 (1.01-4.10)	0.046	2.25 (1.12-4.52)	0.022
PD-L1 expression (CPS ≥10 vs. <10)	0.63 (0.40-1.01)	0.053				
Differentiation						
Moderate vs. Well	1.00 (0.55-1.81)	0.992				
Poor vs. Well	0.64 (0.30-1.37)	0.252				
Cancer type (Postoperative recurrence vs. Primary metastatic)	0.78 (0.49-1.24)	0.286				
Site of primary tumor						

Middle thoracic vs. Upper	1.35 (0.72-2.54)	0.347				
Lower thoracic vs. Upper	1.10 (0.56-2.18)	0.780				
Liver metastases (Yes vs. No)	0.86 (0.43-1.74)	0.679				
Lung metastases (Yes vs. No)	1.35 (0.76-2.37)	0.305				
Number of metastatic sites (≥2 vs. <2)	0.95 (0.55-1.63)	0.844				
Autoantibody (Positive vs. Negative) [*]	0.56 (0.36-0.87)	0.011	0.20 (0.05-0.70)	0.013		
Anti-nuclear antibody (Positive vs. Negative) [*]	0.45 (0.28-0.71)	0.001			0.07 (0.01-0.37)	0.002
Anti-Ro52 antibody (Positive vs. Negative)	0.89 (0.44-1.80)	0.750				
Lines of PD-1 Inhibitor therapy (⩾2 vs. 1)	1.95 (0.97-3.93)	0.063				
PD-1 Inhibitors						
Tislelizumab vs. Camrelizumab	0.99 (0.51-1.93)	0.986				
Toripalimab vs. Camrelizumab	1.10 (0.64-1.90)	0.733				
Sintilimab vs. Camrelizumab	1.37 (0.65-2.93)	0.410				
irAEs (Yes vs. No)	0.47 (0.29-0.76)	0.002	0.57 (0.32-1.00)	0.051	0.64 (0.37-1.09)	0.100

Abbreviations: PFS, Progression-Free Survival; HR, hazard ratio; 95% CI, 95% confidence interval; ECOG, Eastern Cooperative Oncology Group; PD-L1, programmed death ligand 1; PD-1, programmed death receptor 1; irAEs, immune-related adverse events; ANA, anti-nuclear antibody.

[*] Autoantibody and anti-nuclear antibody were included separately in the multivariate analysis to avoid multicollinearity.

[†] Multivariable time-varying coefficient Cox regression showed significant time-varying effects for autoantibody in Model 1 (interaction HR=2.079, 95% CI: 1.055–4.099, *P*=0.035) and ANA in Model 2 (interaction HR=2.854, 95% CI: 1.277–6.379s, *P*=0.011).

[#] Model 1 included ECOG performance status, autoantibody, irAEs, and an autoantibody-by-log(time) interaction term to account for violation of the proportional hazard's assumption.

[$] Model 2 included ECOG performance status, ANA, irAEs, and an ANA-by-log(time) interaction term to account for violation of the proportional hazard's assumption. Separate multivariate models were constructed for ANA and autoantibody to avoid multicollinearity. Time-varying coefficient Cox regression with covariate-by-log(time) interaction was used when the proportional hazards assumption was violated.

Supplementary Table S7. Univariable Cox proportional hazards regression and multivariable time-varying coefficient Cox regression analyses for OS (sensitivity analysis restricted to patients with known PD-L1 expression)

Variables	Univariable		Multivariable [#]	
	HR (95% CI)	*P* value	HR (95% CI)	*P* value
Age, years	1.03 (0.99-1.06)	0.145		
Gender (Female vs. Male)	1.29 (0.65-2.55)	0.461		
ECOG performance status (≥2 vs. 0-1)	3.39 (1.69-6.77)	0.001	4.36 (2.08-9.17)	<0.001
PD-L1 expression (CPS ≥10 vs. <10) [†]	0.52 (0.31-0.88)	0.015	0.15 (0.01-1.89)	0.142
Differentiation				
Moderate vs. Well	1.27 (0.63-2.56)	0.503		
Poor vs. Well	1.60 (0.72-3.57)	0.253		
Cancer type (Postoperative recurrence vs. Primary metastatic)	0.83 (0.50-1.39)	0.487		
Site of primary tumor				
Middle thoracic vs. Upper	2.17 (1.04-4.52)	0.039	1.65 (0.78-3.51)	0.195
Lower thoracic vs. Upper	1.02 (0.46-2.30)	0.956	1.02 (0.44-2.32)	0.971
Liver metastases (Yes vs. No)	0.85 (0.39-1.87)	0.693		
Lung metastases (Yes vs. No)	1.12 (0.60-2.11)	0.723		
Number of metastatic sites (≥2 vs. <2)	0.93 (0.50-1.71)	0.809		
Autoantibody (Positive vs. Negative)	0.62 (0.37-1.02)	0.058		
Anti-nuclear antibody (Positive vs. Negative) [†]	0.54 (0.32-0.90)	0.018	0.03 (0.001-0.44)	0.012
Anti-Ro52 antibody (Positive vs. Negative)	1.07 (0.53-2.18)	0.848		
Lines of PD-1 Inhibitor therapy (⩾2 vs. 1)	1.21 (0.55-2.65)	0.643		

PD-1 Inhibitors				
Tislelizumab vs. Camrelizumab	1.06 (0.51-2.23)	0.873		
Toripalimab vs. Camrelizumab	1.31 (0.71-2.42)	0.395		
Sintilimab vs. Camrelizumab	1.61 (0.69-3.79)	0.275		
irAEs (Yes vs. No)	0.61 (0.37-1.01)	0.056		

Abbreviations: OS, Overall Survival; HR, hazard ratio; 95% CI, 95% confidence interval; ECOG, Eastern Cooperative Oncology Group; PD-L1, programmed death ligand 1; PD-1, programmed death receptor 1; irAEs, immune-related adverse events; ANA, anti-nuclear antibody.

† In the multivariable time-varying coefficient Cox model, a significant time-varying effect was observed for anti-nuclear antibody positivity (interaction HR = 3.487, 95% CI: 1.165–10.439, P = 0.026), whereas no significant time-varying effect was observed for PD-L1 expression (interaction HR = 1.631, 95% CI: 0.615–4.323, P = 0.325).

# PD-L1 expression and ANA status violated the proportional hazards assumption and were modeled using time-varying coefficients with covariate-by-log(time) interaction terms. Separate multivariate models were constructed for ANA and autoantibody to avoid multicollinearity. Time-varying coefficient Cox regression with covariate-by-log(time) interaction was used when the proportional hazards assumption was violated.

Supplementary Table S8. TNM characteristics according to pretreatment ANA status

A. Patients without prior surgery: pretreatment clinical TNM classification

Variable, n (%)	Total (N = 118)	ANA-negative (n = 76)	ANA-positive (n = 42)	*P* value
cT category				0.543
T2	4 (3.39)	3 (3.95)	1 (2.38)	
T3	76 (64.41)	46 (60.53)	30 (71.43)	
T4	38 (32.20)	27 (35.53)	11 (26.19)	
cN category				0.416
N1	21 (17.80)	16 (21.05)	5 (11.90)	
N2	50 (42.37)	32 (42.11)	18 (42.86)	
N3	47 (39.83)	28 (36.84)	19 (45.24)	
cM category				0.238
M0	62 (52.54)	43 (56.58)	19 (45.24)	
M1	56 (47.46)	33 (43.42)	23 (54.76)	
Clinical stage group				0.461
Stage III	24 (20.34)	17 (22.37)	7 (16.67)	
Stage IV	94 (79.66)	59 (77.63)	35 (83.33)	

B. Patients with postoperative recurrence: rTNM classification

Variable, n (%)	Total (N = 62)	ANA-negative (n = 38)	ANA-positive (n = 24)	*P* value
pT category				0.864
T1	3 (4.84)	2 (5.26)	1 (4.17)	
T2	11 (17.74)	8 (21.05)	3 (12.50)	
T3	47 (75.81)	27 (71.05)	20 (83.33)	
T4	1 (1.61)	1 (2.63)	0 (0.00)	
pN category				0.649
N0	11 (17.74)	5 (13.16)	6 (25.00)	
N1	30 (48.39)	20 (52.63)	10 (41.67)	
N2	15 (24.19)	9 (23.68)	6 (25.00)	
N3	6 (9.68)	4 (10.53)	2 (8.33)	
cM status at recurrence				—

M1	62 (100.00)	38 (100.00)	24 (100.00)	

Data are presented as n (%). Percentages were calculated using the total number of patients in each column within the corresponding clinical setting. For patients without prior surgery, cT, cN, cM, and clinical stage group refer to assessments before initiation of the index PD-1 inhibitor-based immunochemotherapy regimen. For patients with postoperative recurrence, pT and pN refer to the original surgical pathology. M0 refers to the absence of distant metastasis at the original surgery and does not describe metastatic status at recurrence. P values were calculated using the Fisher–Freeman–Halton exact test for cT, pT, and pN and Pearson's chi-square test without continuity correction for cN, cM, and clinical stage group. No comparison was performed for M status at initial surgery because all patients in this subgroup were M0. ANA, antinuclear antibody; TNM, tumor–node–metastasis; c, clinical; p, pathological.

Supplementary Table S9. Treatment characteristics according to pretreatment ANA status

Characteristic	Total (N = 180)	ANA-negative (n = 114)	ANA-positive (n = 66)	*P* value
PD-1 inhibitor, n (%)				0.977
Camrelizumab	64 (35.56)	40 (35.09)	24 (36.36)	
Tislelizumab	28 (15.56)	17 (14.91)	11 (16.67)	
Toripalimab	53 (29.44)	34 (29.82)	19 (28.79)	
Sintilimab	35 (19.44)	23 (20.18)	12 (18.18)	
Chemotherapy regimen, n (%)				0.776
Nab-paclitaxel + carboplatin	82 (45.56)	51 (44.74)	31 (46.97)	
Nab-paclitaxel + lobaplatin	42 (23.33)	29 (25.44)	13 (19.70)	
Nab-paclitaxel + nedaplatin	38 (21.11)	24 (21.05)	14 (21.21)	
Nab-paclitaxel + cisplatin	18 (10.00)	10 (8.77)	8 (12.12)	

Data are presented as n (%). Percentages were calculated using the total number of patients in each column as the denominator. P values represent overall comparisons of the PD-1 inhibitor and chemotherapy regimen distributions between the ANA-negative and ANA-positive groups and were calculated using Pearson's chi-square test. Reliable patient-level counts of PD-1 inhibitor and chemotherapy cycles actually administered were unavailable and are therefore not reported. ANA, antinuclear antibody; PD-1, programmed cell death protein 1; nab-paclitaxel, nanoparticle albumin-bound paclitaxel.
